# A novel semiautomated method for background activity and biological tumour volume definition to improve standardisation of ^18^F-FET PET imaging in glioblastoma

**DOI:** 10.1186/s40658-022-00438-2

**Published:** 2022-02-05

**Authors:** Caterina Brighi, Simon Puttick, Shenpeng Li, Paul Keall, Katherine Neville, David Waddington, Pierrick Bourgeat, Ashley Gillman, Michael Fay

**Affiliations:** 1 ACRF Image X Institute, Sydney School of Health Sciences, Faculty of Medicine and Health, The University of Sydney, Sydney, Australia; 2 Australian e-Health Research Centre, Commonwealth Scientific and Industrial Research Organization, Royal Brisbane and Women’s Hospital, Brisbane, Australia; 3 GenesisCare, Newcastle, Australia; 4 School of Medicine and Public Health, The University of Newcastle, Newcastle, Australia

**Keywords:** ^18^F-FET PET, Glioma, PET imaging, Method standardisation, Background activity

## Abstract

**Background:**

Multicentre clinical trials evaluating the role of ^18^F-Fluoroethyl-l-tyrosine (^18^F-FET) PET as a diagnostic biomarker in glioma management have highlighted a need for standardised methods of data analysis. ^18^F-FET uptake normalised against background in the contralateral brain is a standard imaging technique to delineate the biological tumour volume (BTV). Quantitative analysis of ^18^F-FET PET images requires a consistent and robust background activity. Currently, defining background activity involves the manual selection of an arbitrary region of interest, a process that is subject to large variability. This study aims to eliminate methodological errors in background activity definition through the introduction of a semiautomated method for region of interest selection. A new method for background activity definition, involving the semiautomated generation of mirror-image (MI) reference regions, was compared with the current state-of-the-art method, involving manually drawing crescent-shape (gCS) reference regions. The MI and gCS methods were tested by measuring values of background activity and resulting BTV of ^18^F-FET PET scans of ten patients with recurrent glioblastoma multiforme generated from inputs provided by seven readers. To assess intra-reader variability, each scan was evaluated six times by each reader. Intra- and inter-reader variability in background activity and BTV definition was assessed by means of coefficient of variation.

**Results:**

Compared to the gCS method, the MI method showed significantly lower intra- and inter-reader variability both in background activity and in BTV definition.

**Conclusions:**

The proposed semiautomated MI method minimises intra- and inter-reader variability, providing a valuable approach for standardisation of ^18^F-FET PET quantitative parameters.

*Trial registration* ANZCTR, ACTRN12618001346268. Registered 9 August 2018, https://www.anzctr.org.au/Trial/Registration/TrialReview.aspx?id=374253

**Supplementary Information:**

The online version contains supplementary material available at 10.1186/s40658-022-00438-2.

## Background

The important role of ^18^F-Fluoroethyl-l-tyrosine (^18^F-FET) PET imaging in the management of glioma patients is becoming widely recognised around the world by associations such as the European Association of Neuro Oncology, the European Association of Nuclear Medicine (EANM) and the Response Assessment in Neuro Oncology working group [1–4]. ^18^F-FET PET imaging has proven useful in the diagnosis of primary tumour lesions (where biopsy may not be possible), the differentiation between chemoradiation-related changes and tumour recurrence at follow-up, the assessment of response to treatment with certain anticancer drugs and patients’ prognosis [1]. Despite the increasing evidence that ^18^F-FET PET imaging improves management of glioma patients, there remain critical limitations hampering its inclusion into clinical practice. One barrier to increased use of ^18^F-FET PET is the prevalence of small single-centre studies or studies based on retrospective PET data collection, which has thus far prevented a robust validation of the clinical value of ^18^F-FET PET as an imaging biomarker [1]. Consequently, there has been an emerging interest from the neuro-oncology community in combining efforts to validate the utility of ^18^F-FET PET as an imaging biomarker in prospective multicentre clinical trials, such as the current TROG 18.06 trial [5].

As multicentre trials emerge, there is a need to standardise methods for data acquisition and analysis, to enable correlations and comparisons of results from different sites. Evaluation metrics of such trials are often determined via PET tracer uptake measures, such as standard uptake values (SUV) and tumour-to-brain ratio (TBR), where TBR is defined as the ratio between activity in the tumour lesion and activity in a background reference region in the healthy, contralateral part of the brain. In the current literature on published ^18^F-FET PET studies, there is large variability in reported values of SUV and TBR taken as threshold for differentiation between tumour and non-tumour tissue [6]. Variability of SUV values between studies arises not only from the acquisition of scans at different timepoints, but also from patients’ physiological factors that can affect ^18^F-FET SUV in various brain regions, and thereby affect the quantification of ^18^F-FET uptake in brain tumours [7]. Thus, due to this large intra-patient variability in ^18^F-FET SUV values, TBR values are a preferred evaluation metric in the intra- and inter-individual comparison of PET results [6]. However, TBR values are also variable in the literature and in clinical practice, and their variability arises from the intra- and inter-reader variability in the selection of the background reference region. Despite the EANM guidelines for brain imaging highlight that the choice of background reference region is a critical step for the reliable quantification of TBR measurements [8], there is still no standard procedural recommendation for this process. Thus, inconsistent approaches are found in the current literature and in clinical practice, mostly involving the arbitrary definition of a 2D circular region of interest or a 3D spherical volume of interest (VOI) in the contralateral hemisphere including grey and white matter [9–17]. A recent study by Unterrainer et al. [6] has demonstrated that adding guidelines to the definition of a crescent-shaped VOI in the contralateral hemisphere including grey and white matter significantly reduces intra- and inter-reader variability in the measurement of reference background activity, thus providing a first important step towards the standardisation of background activity assessment for clinical application. However, this method still requires significant manual reader input, which remains time-consuming and a source of methodological error. This variability in reference regions definition is then reflected in the variability in values of background SUV and, consequently, in biological tumour volume (BTV) delineation, which is based on TBR threshold-based segmentation [6]. The resulting uncertainty in BTV definition impacts several therapeutic decision-making processes, ranging from radiotherapy treatment planning to the assessment of treatment response.

In this study, we present a semiautomated method for the generation of background reference regions in the contralateral hemisphere. We show that this semiautomated method improves on gold standard techniques by minimising intra- and inter-reader variability, substantially reducing the time spent by the reader on manual contouring and accounting for tumour size and specific location within the brain. This method encompasses the automated generation of a mirror-image (MI) VOI in the contralateral hemisphere with respect to the anterior–posterior midline reflecting the size, shape and location of the tumour. The strength of this method is that the selection of the size and location of the reference region is not arbitrary, but it reflects the characteristics of the tumour lesion. We aim to assess the efficiency of the MI method by comparing the manual time spent by the reader and the intra- and inter-reader variability in measurements of background SUV and BTV obtained with this method versus that obtained with the guided crescent-shape (gCS) VOI method published by Unterrainer et al. [6]. We selected the gCS as the standard method of comparison because it is the manual method with the lowest intra- and inter-reader variability reported in recent literature [6]. We hypothesize that the MI method will lead to significantly lower values of variability in background mean SUV (SUV_mean_) and BTV and reduced manual reader input time compared to the gCS method.

## Methods

### Clinical trial information

This study was a diagnostic, non-randomised, uncontrolled, open-label, single-centre, single-arm, bioavailability, pilot clinical trial, enrolling recurrent glioblastoma multiforme (GBM) patients. Adult patients were eligible for inclusion if they had previously histologically confirmed GBM at resection, progression noted on pre-enrolment MRI scan and an Eastern Cooperative Oncology Group performance status score of ≤ 2. Exclusion criteria were pregnancy, lactation and residence geographically remote from the treating centre. All patients enrolled in the study provided written informed consent in accordance with institutional guidelines. Ethics approval for this study was obtained from the Bellberry Human Research Ethics Committee in August 2018 (Ethics approval number: 2017-11-885). Recruitment for the trial (registration No./date: ACTRN12618001346268/09-08-2018) started in October 2018 and was completed in October 2021, with a total enrolment of ten patients. Details of the data acquisition protocols are available on the trial registration page [18].

### Data, readers and manual input objects

For this study ^18^F-FET PET data of ten patients with recurrent GBM obtained as part of the Genesis GBM 001 clinical trial were used. Image pre-processing steps included conversion of values of activity into SUV, registration of PET images with CT images, and brain extraction. If severe head rotation was observed in the axial view, a rigid rotation transformation was applied to align the head positions with the image’s orthogonal coordinates before generating brain-extracted image. Complete details of imaging method and image pre-processing steps are included in Additional file 1. Seven readers, including five researchers in medical imaging with at least three years of experience in PET image analysis (C.B., S.P., S.L., P.B., A.G.) and two radiation oncologists (M.F., K.N.), were involved in providing manual inputs for this study. The readers were provided with brain-extracted ^18^F-FET PET images of the patients in units of SUV and an instruction manual for the generation of the required manual input objects for each of the two methods (MI and gCS). The manual input objects required from each reader on each dataset for the gCS method included the coordinates (x,y,z) of a seed located within the main tumour lesion (region of high ^18^F-FET PET SUV) and a crescent-shape VOI manually defined according to guidelines as per Unterrainer et al. [6]. Conversely, the manual input objects for the MI method only included the coordinates of a seed located within the main tumour lesion. To enable evaluation of intra-reader variability, each reader provided six repeats of the manual input objects for each patient's dataset.

### Segmentation methods

#### Mirror-image method

**Fig. 1:**
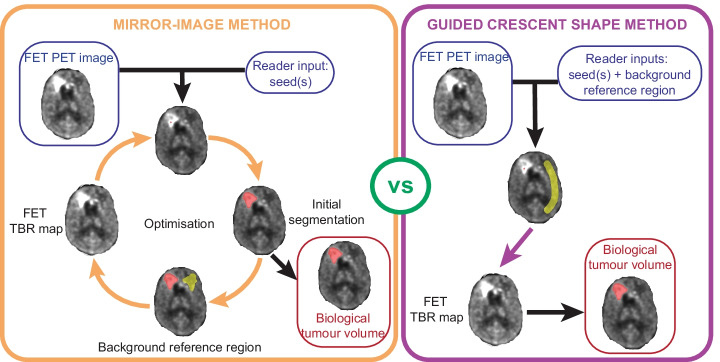
Schematics of the algorithm for the two methods. Mirror-image method involving an iterative optimisation process. Guided crescent-shape method involving a linear process. FET = ^18^F-Fluoroethyl-l-tyrosine; TBR = tumour-to-brain ratio

The algorithm for the MI method for background contralateral reference (CTRL) VOI definition was developed in Python and is available on GitHub at the following link https://github.com/cbri92/FETsegmenter.git. The ^18^F-FET PET brain-extracted image and the coordinates of the defined seed within the ^18^F-FET-enhancing lesion are used to develop an initial segmentation of the ^18^F-FET-enhancing tumour lesion (BTV_0_) on the ^18^F-FET PET image by use of a region-growing algorithm and a FET SUV threshold of 2.2. This value has been previously determined as a cut-off threshold for identification of recurrent glioma [19, 20]. This initial BTV_0_ is used to automatically generate a MI VOI in the contralateral hemisphere (CTRL_0_ VOI), excluding potential overlapping areas. The SUV_mean_ calculated in the CTRL_0_ VOI is then used to normalise the ^18^F-FET PET image and generate a FET TBR map. The FET TBR map is then used as new input image for growing a new BTV from the input seed coordinates, this time with a FET TBR threshold set at 1.9. This threshold value was chosen based on literature demonstrating the utility of this value in identifying tumour recurrence and progression [21, 22]. The process is repeated in a loop until the convergence condition is reached. The convergence condition set is that the volume of the BTV defined on the FET TBR map equals the volume of the generated CTRL VOI from the previous iteration ± 0.2 cm^3^. Once convergence is reached, any volume of the CTRL VOI overlapping with the BTV is removed from the CTRL VOI final segmentation, hence excluding infiltrating tumour tissue from the selected background reference region. Then, the SUV_mean_ in the MI CTRL_MI_ VOI and the volume of the BTV_MI_ are extracted for statistical analysis. A schematic representation of the algorithm pipeline is illustrated in Fig. 1. This method accounts for the presence of multiple tumour lesions (defined as multiple seeds by the reader) in the generation of the FET TBR map and, consequently, of the BTV_MI_.

#### Guided crescent-shape method

The generation of the CTRL VOI and BTV with the guided crescent-shape method requires as inputs the ^18^F-FET PET brain-extracted image, the coordinates of the reader-defined seed and the crescent-shape VOI manually drawn by the reader (CTRL_CS_ VOI). First, the SUV_mean_ in the CTRL_CS_ VOI is calculated and used to normalise the ^18^F-FET PET brain-extracted image to generate a FET TBR map. Then, the segmentation of the BTV_CS_ is developed on the FET TBR map by use of a region-growing algorithm and a FET TBR threshold of 1.9, based on previous studies and consistent with the MI method [21, 22]. For this method no optimisation is performed and the SUV_mean_ in the crescent-shape CTRL_CS_ VOI and the volume of the BTV_CS_ are directly extracted for statistical analysis. A schematic representation of the pipeline used for the crescent-shape method is illustrated in Fig. 1. This method does not account for the presence of multiple tumour lesions in the generation of the FET TBR map and, consequently, of the BTV_CS_, as FET TBR map generation is solely determined by the manually drawn CTRL_CS_ VOI defined by the reader.

### Evaluation metrics

The parameters used to compare the variability between the two studies include the SUV_mean_ in the CTRL VOI and the volume of BTV. For these two parameters variability is assessed by calculating the coefficient of variation (CoV), defined as the ratio between the standard deviation and the mean value of the parameter. Intra-reader variability is defined as the individual CoV of the parameter’s value obtained from the six repeats of a particular scan. Inter-reader variability is defined as the CoV of the mean value of the parameter obtained from the seven readers regarding a particular scan. Intra- and inter-reader reliability are also evaluated via intraclass correlation coefficient (ICC). ICC estimates and their 95% confident intervals are calculated in Python using the pingouin statistical package based on two-way random-effects, absolute-agreement, single rater/measurement model [23]. ICC values < 0.5 indicate poor reliability, values 0.5–0.75 indicate moderate reliability, values 0.75–0.9 indicate good reliability and values > 0.90 indicate excellent reliability [24]. The time taken for the definition of the manual inputs from each reader and the time taken by the algorithm to generate a BTV from the readers’ inputs are also reported for both methods.

### Statistical analysis

The statistical analysis performed to assess intra-reader variability between the MI and the gCS methods is a two-tailed, matched-pairs Wilcoxon signed rank test, *α* = 0.05, between values of CoV for each parameter for each reader. Additionally, overall group comparison on intra-reader variability is assessed by combining the CoV of a parameter from each reader and performing a two-tailed, Mann Whitney unpaired U test, *α* = 0.05. The statistical analysis performed to assess inter-reader variability between the MI and the gCS methods is a two-tailed, matched-pairs Wilcoxon signed rank test, *α* = 0.05, between values of CoV for each parameter obtained from all the readers.

## Results

### Guided crescent-shape VOI

The use of the gCS method for background activity assessment resulted in a median intra-reader CoV of 1.72% (range 0.34–9.99%) for CTRL SUV_mean_ and 6.77% (range 0–65.23%) for BTV, and in a median inter-reader CoV of 2.80% (range 1.00–4.35%) for CTRL SUV_mean_ and 14.37% (range 5.03–36.30%) for BTV (Tables 1 and 2). The average time spent by each reader for the generation of the manual inputs required for this method for a single repeat of one patient was 138 s, and the time taken by the algorithm to generate a BTV from the reader inputs for a single repeat of one patient was 17 s (Table 3).

**Table 1:** Intra- and inter-reader coefficient of variation of the CTRL SUV_mean_

CoV CTRL SUV_mean_	Mirror-image [median (range)]	Guided crescent-shape [median (range)]	*p* value
Intra-reader variability			
Overall group	0% (0–2.15%)	1.72% (0.34–9.99%)	< 0.0001
Reader #1	0% (0–0.26%)	0.99% (0.34–4.85%)	0.002
Reader #2	0% (0–2.15%)	1.69% (0.53–4.84%)	0.004
Reader #3	0% (0–1.62%)	1.25% (0.56–6.13%)	0.004
Reader #4	0% (0–1.33%)	1.10% (0.44–4.22%)	0.004
Reader #5	0% (0–0.25%)	1.73% (0.47–3.62%)	0.002
Reader #6	0% (0–1.83%)	3.94% (1.07–9.99%)	0.002
Reader #7	0% (0–0.05%)	3.10% (1.06–7.92%)	0.002
Inter-reader variability			
Overall group	0.005% (0–1.05%)	2.80% (1.00–4.35%)	0.002

**Table 2:** Intra- and inter-reader coefficient of variation of the BTV

CoV BTV	Mirror-image [median (range)]	Guided crescent-shape [median (range)]	*p* value
Intra-reader variability			
Overall group	0% (0–3.88%)	6.77% (0–65.23%)	< 0.0001
Reader #1	0% (0–2.51%)	3.02% (1.33–48.74%)	0.002
Reader #2	0% (0–2.51%)	8.36% (2.16–32.16%)	0.002
Reader #3	0% (0–0.65%)	6.73% (1.62–18.28%)	0.002
Reader #4	0% (0–0.52%)	4.14% (0.70–52.65%)	0.002
Reader #5	0% (0–0.65%)	5.30% (2.20–21.56%)	0.002
Reader #6	0% (0–3.88%)	13.72% (0–65.23%)	0.004
Reader #7	0% (0–0.66%)	12.91% (3.96–31.97%)	0.002
Inter-reader variability			
Overall group	0.05% (0–36.00%)	14.37% (5.03–36.30%)	0.002

**Table 3:** Time taken for manual input generation and BTV computation for a single repeat [average/median (range)]

Time required for task	Mirror-image	Guided crescent-shape
Manual inputs generation	54 s/30 s (15 s-150 s)	138 s/150 s (75 s-210 s)
BTV computation	54 s/29 s (22 s-184 s)	17 s/15 s (7 s-35 s)

### Mirror-image VOI

The use of the MI method for background activity assessment resulted in a median intra-reader CoV of 0% (range 0–2.15%) for CTRL SUV_mean_ and 0% (range 0–3.88%) for BTV, and in a median inter-reader CoV of 0.005% (range 0–1.05%) for CTRL SUV_mean_ and 0.05% (range 0–36.00%) for BTV (Tables 1 and 2). The time spent by each reader for the generation of the manual inputs required for this method for a single repeat of one patient was 54 s, and the time taken by the algorithm to generate a BTV from the reader inputs for a single repeat of one patient was 54 s (Table 3).

### Comparison of background assessment methods

**Fig. 2:**
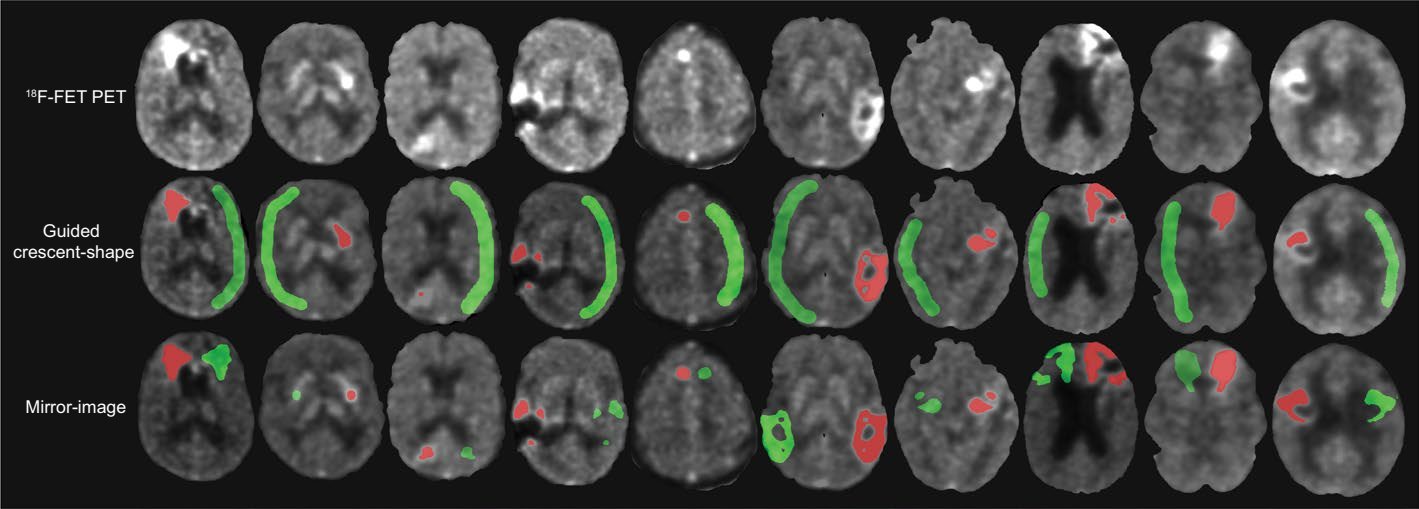
Representative images and segmentations obtained with the two background assessment methods. Top–bottom: ^18^F-FET PET images of ten GBM patients; ^18^F-FET TBR images with overlays of BTV (red) and CTRL VOI (green) obtained with the gCS method; ^18^F-FET TBR images with overlays of BTV (red) and CTRL VOI (green) obtained with the MI method. BTV = biological tumour volume; CTRL = contralateral background reference region; FET = ^18^F-Fluoroethyl-l-tyrosine; TBR = tumour-to-brain ratio; VOI = volume of interest

**Fig. 3:**
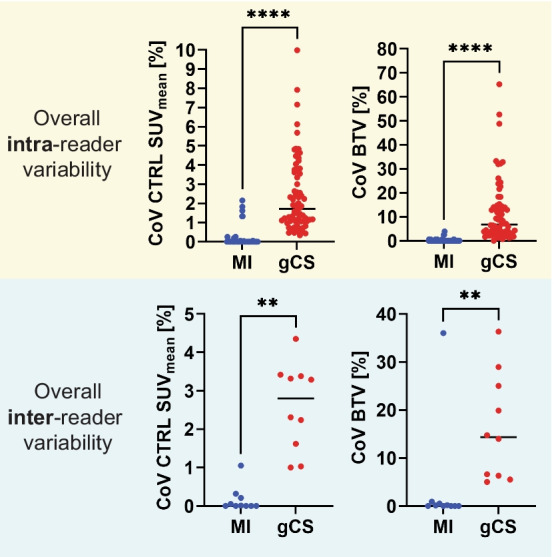
Summary plots of intra-reader and inter-reader coefficient of variation (CoV) for the overall group. The plots show the comparison of the intra-reader and inter-reader CoV of the CTRL SUV_mean_ (top row) and BTV (bottom row) between the MI (blue) and the gCS (red) methods for the overall group of readers. *p < 0.05, **p < 0.01, ***p < 0.001, ****p < 0.0001, ns = no significant difference. BTV = biological tumour volume; CoV = coefficient of variation; CTRL = contralateral background reference region; gCS = guided crescent-shape; MI = mirror-image; SUV = standard uptake value

An example of the background reference regions obtained with both methods is shown in Fig. 2. The comparison between MI and gCS methods reveals that all the readers obtained significantly different (*p* < 0.001) mean values of CTRL SUV_mean_ and BTV for almost all datasets (Additional file 1: Fig. S1). While no clear trend was established, for eight of the ten datasets the gCS method resulted in higher estimates of CTRL SUV_mean_ and, consequently, lower estimates of BTV than the MI method (Additional file 1: Fig. S2). Additionally, for each dataset the mean values of CTRL SUV_mean_ and BTV were more consistent across readers when determined via MI method rather than gCS method (Additional file 1: Fig. S2). When considering variability metrics, all readers obtained significantly lower values of intra- and inter-reader CoV both for CTRL SUV_mean_ (*p* < 0.0001 and *p* = 0.002, respectively; Fig. 3 and Additional file 1: Fig. S3) and BTV (*p* < 0.0001 and *p* = 0.002, respectively; Fig. 3 and Additional file 1: Fig. S3) by use of MI method. While intra- and inter-reader ICC values revealed excellent reliability in estimates of BTV and CTRL SUV_mean_ with both methods, the MI method resulted in higher ICC values than the gCS (Additional file 1: Fig. S4, Tables S1, S2). The average time spent by each reader in generating inputs for the MI method was ~ 2.5 times faster than for the gCS method (Table 3).

## Discussion

The adoption of a consistent method for the standardisation of background activity definition in ^18^F-FET PET imaging is needed to ensure the reproducible and reliable quantification of ^18^F-FET uptake parameters, which is necessary for the comparison of multicentre ^18^F-FET PET clinical trials. Previous studies show that the intra- and inter-reader variability associated with current, most common methods of selecting regions of background activity (2D circular region of interest or 3D spherical VOI) result in background SUV changes of up to ± 8% [6]. This large variability has been mostly attributed to the variable size and insufficient inclusion of different types of tissue within the selected 2D reference region, and to the imprecise and arbitrary selection of the positioning of the 3D reference region, which could result in the inclusion of areas with notably higher ^18^F-FET uptake, such as venous structures and areas of grey matter [25]. The use of a crescent-shape VOI has been accepted and recommended by the joint EANM/EANO/RANO practice guidelines/SNMMI procedure standards as a solution to these issues, as this method allows for the inclusion in the reference region of a larger volume of tissue from both white and grey matter, and for the morphological adaptation of the reference region such that to exclude ventricles and venous sinuses [26]. Unterrainer et al. [6] were able to demonstrate that when the readers are given a set of rules to draw the crescent-shape VOI, the variability in values of background SUV is significantly reduced compared to the other methods, regardless of the level of experience of the reader performing the task. In our study, we were able to reproduce similar values of intra- and inter-reader CoV for the CTRL SUV_mean_ obtained by use of the gCS method to the values reported by Unterrainer et al. [6], i.e. group intra-reader CoV median 1.10% (range 0.52–2.36%) and group inter-reader CoV median 1.19% (range 0.84–1.89%). However, when we investigated how this variability would propagate in the definition of the BTV, our results demonstrated a substantially higher intra-reader CoV with changes up to 65%. In clinical practice this large dispersion associated with the delineation of BTV can significantly impact several important decision-making processes, such as the ability to assess response to chemotherapy or antiangiogenic therapy, where changes > 20% in BTV are considered treatment response, or in radiotherapy treatment planning, where the BTV is used to delineate areas that should receive a dose-boost [27–31].

In this study, we validated a semiautomated method that could eliminate the variability in background activity assessment, with the aim to also eliminate, or at least minimise, the variability in BTV definition compared to the gCS method. We propose the MI method, which involves the semiautomated generation of a background reference volume as mirror-image of the tumour volume, as a robust method lacking any arbitrary interpretation. Our analysis showed that using the MI method to generate background reference regions not only resulted in a significant reduction in intra- and inter-reader variability in values of CTRL SUV_mean_ compared to the gCS method (with median values of CoV of 0%), but also minimised variability in BTV definition. As mentioned above, this is critically important for a series of clinical decision-making processes that rely on precise BTV estimates, such as assessment of treatment response and radiotherapy treatment planning. The significant reduction in values of CoV, which had median values of 0% for both CTRL SUV_mean_ and BTV, is mainly due to the elimination of biases involved in the individual selection of placement of the background reference region, but also to the consideration of the size and percentage of involvement of different tissue types and brain structures which are reflected in the tumour lesion. Taking into account the size and the relative involvement of different tissue types in the background reference region is particularly important as recent studies have demonstrated that selected normal brain structures have intrinsically high physiological ^18^F-FET uptake, and that patient-specific factors, such as gender and body mass index, can affect values of ^18^F-FET uptake in the brain in a patient-specific manner [7, 25]. Consequently, if the tumour is small and located in an area of the brain with intrinsically high ^18^F-FET uptake, selecting a large background reference VOI from other areas of healthy brain might result in an underestimation of the background reference SUV and, in turn, in an overestimation of TBR values. With the MI method these subjective and local variations in ^18^F-FET uptake are appropriately normalised. Additionally, the MI method is the first method to include different areas of background activity in the final CTRL_MI_ VOI for cases of patients with multiple tumour lesions located in different areas of the brain. This is not accounted for in current methods that involve the selection of a single contiguous VOI.

A possible concern associated with the use of the MI method is the potential for tumour cells to infiltrate the contralateral brain. This concern has been addressed with the implementation of the algorithm used to generate the CTRL_MI_ VOI, that excludes any area of ^18^F-FET uptake above background from the final reference region. This solution was developed under the assumption that the potential inclusion of any areas of infiltrating tumour with similar ^18^F-FET uptake to the surrounding healthy brain tissue in the final CTRL_MI_ VOI would not affect the overall CTRL SUV_mean_ value and BTV definition.

Overall, the MI method represents the first semiautomated method resulting in median 0% variability both on the measurement of values of CTRL SUV_mean_ and BTV. The utility of this method for research applications is clear, as it provides a means not only to evaluate ^18^F-FET quantitative parameters reliably and reproducibly, thereby facilitating the assessment of multicentre clinical trials, but also to perform objective derivations of imaging features used to build ^18^F-FET PET-based predictive models. However, this method could also be particularly useful in the clinical setting, where it would be used to semiautomatically generate an initial BTV in a much shorter time and more consistent manner than the gCS method, with the assumption that the resulting BTV would then require minimal final adjustments from the physician. Fully automated approaches for segmentation of solid tumours on PET images have also been shown to reduce inter-reader variability, without, however, being able to provide valid and plausible segmentations of all tumours [32]. This is due to the intrinsic limitation of any fully automated algorithm to account for clinical information absent in the PET image, such as knowledge of patient-specific high uptake regions, which could be incorrectly identified as the tumour location by the algorithm [33]. As such, the clinical consensus recognises that standardised technical approaches for glioma PET imaging procedures need active physician involvement in the segmentation process, particularly for the initial selection of the tumour location and in the review of the final contours [33]. Our MI method satisfies this requirement, with a simple and transparent algorithm that can be integrated into commercial treatment planning systems.

Furthermore, while in this study we validated the use of the MI method for the generation of reproducible ^18^F-FET quantitative parameters for recurrent GBM patients, the use of this method could be expanded to newly diagnosed GBM cases with the appropriate selection of ^18^F-FET SUV and TBR threshold values. Finally, the application of this method could be expanded for the standardisation of other types of PET images used in neuro-oncology, such as ^18^F-FMISO, ^18^F-FDOPA, ^18^F-FDG, but also for quantification of PET images used in the diagnosis of other neurodegenerative diseases, such as epilepsy and stroke.

A limitation of this study is that the MI method might not be suitable for patients with tumour lesions heavily involving areas of the brain along the anterior–posterior midline, such as the corpus callosum, patients where tumour growth has severely compromised the anatomical symmetry in the contralateral lobe or patients with multifocal bilateral diseases. While these patients represent only a small percentage (~ 10%) of GBM cases seen in clinical practice, developing an automated method for the generation of representative background reference regions for this subgroup of patients should be the focus of future research work.

Another limitation of this study is the lack of a statistical comparison between expert (nuclear medicine physicists, radiation oncologists) and non-expert readers (medical imaging researchers with experience in PET imaging processing). In this regard, we built on the results published by Unterrainer et al. [6], which demonstrated the lack of significant difference in variability of ^18^F-FET quantitative parameters derived with the gCS method between expert and unexpert readers.

Finally, a limitation of this study is the inclusion of only ten patients’ datasets, which correspond to the number of datasets available from patients who have enrolled in this trial. While this limitation does not affect measurements of inter-reader variability, it could impact results of intra-reader variability. It is worth mentioning that intra-reader variability does not have as big of an impact on routine clinical decisions as does inter-reader variability, as the segmentation of a single patient’s data is normally done only once by a single physician, but it can be repeated by multiple physicians for credentialing. However, for sake of completeness, we suggest that future studies should validate this method on a larger sample dataset.

Future work should focus on evaluating the use of the MI method for the analysis of prospective multicentre clinical trials foreseeing the recruitment of large sample datasets, such as the current TROG 18.06 trial [5].

## Conclusions

In conclusion, this study demonstrated that using the semiautomated method of generating mirror-image VOI in the contralateral hemisphere for the assessment of background activity in ^18^F-FET PET leads to a fast, reliable and reproducible way of deriving ^18^F-FET PET quantitative parameters. This method could have critical impact in future ^18^F-FET PET imaging research studies, as it would provide clinicians and researchers in the field of nuclear medicine with a standardised and robust way of selecting a background reference region, thereby facilitating the comparability of ^18^F-FET PET studies performed at different centres. Additionally, the MI method could find useful applications in routine clinical practice as a robust tool for the more reproducible definition of BTV segmentations, thus helping clinicians reducing bias in diagnostics evaluations and in making treatment decisions.

## Supplementary Information

**Additional file 1**. Supplemental methods of image acquisition and pre-processing, and supplemental figures and tables of results of comparison of background assessment methods.

## Data Availability

The data that support the findings of this study are available from GenesisCare but restrictions apply to the availability of these data, which were used under license for the current study, and so are not publicly available. Data are however available from the authors upon reasonable request and with permission of GenesisCare.

## Abbreviations

BTV: Biological tumour volume
CTRL: Background contralateral reference
CoV: Coefficient of variation
^18^F-FET: ^18^F-Fluoroethyl-l-tyrosine
GBM: Glioblastoma multiforme
gCS: Guided crescent-shape
ICC: Intraclass correlation coefficient
MI: Mirror-image
SUV: Standard uptake value
TBR: Tumour to brain ratio
VOI: Volume of interest

## Code availability

Code for the generation of the MI and gCS segmentation outputs from reader’s input is publicly available on GitHub at the following link https://github.com/cbri92/FETsegmenter.git.
