# Supplementary material for: A novel semiautomated method for background activity and biological tumour volume definition to improve standardisation of 18F-FET PET imaging in glioblastoma
Source: EJNMMI Phys. 2022 Feb 5;9:9. doi: 10.1186/s40658-022-00438-2 (PMC8818070; doi:10.1186/s40658-022-00438-2)
Supplement: Supplementary file 1 — Additional file 1. Supplemental methods of image acquisition and pre-processing, and supplemental figures and tables of results of comparison of background assessment methods. [file 40658_2022_438_MOESM1_ESM.docx]

**Supplementary Information**

**TITLE**: A novel semiautomated method for background activity and biological tumour volume definition to improve standardisation of ^18^F-FET PET imaging in glioblastoma

**AUTHORS**: Caterina Brighi*, Simon Puttick, Shenpeng Li, Paul Keall, Katherine Neville, David Waddington, Pierrick Bourgeat, Ashley Gillman, Michael Fay

***CORRESPONDENCE**: [caterina.brighi@sydney.edu.au](mailto:caterina.brighi@sydney.edu.au)

**Supplementary method**

*Imaging method*

Doses of radiolabelled ^18^F-FET were purchased from Cyclotek. Radiochemical yield and purity were determined by TLC and HPLC. Doses were administered if the radiochemical purity was > 95%. PET-CT images were acquired 20 min post intravenous injection of ^18^F-FET, using a Clinical PET-CT system (Siemens Biograph mCT). A 10 min PET image was acquired followed by a CT image (acquisition parameters: 3mm slice (64x0.6 mm), pitch 0.8, kV 120) for attenuation correction and co-registration to the MRI data. The PET and CT images were reconstructed using reconstruction algorithms in the software version PET syngo VE60A integrated in the Clinical scanner package (Siemens Biograph mCT) correcting for attenuation and ^18^F detector efficiency. Corrected PET reconstruction parameters: TrueX+TOF (ultraHD-PET), iterations 2, subsets 21, Gaussian filter, FWHM 2 mm; uncorrected PET reconstruction parameters: Iterative TOF, iterations 3, subsets 21, Gaussian filter, FWHM 2 mm; ACCT Brain 3.0 I30f3 reconstruction algorithm: I30f medium smooth 3 mm slice, increment 2 mm, window cerebrum. CT Brain 2.0I31f3 reconstruction algorithm: I31f medium smooth, 2 mm slice, increment 1 mm, window cerebrum.

*Image* pre-processing

DICOM images were converted into NIFTI format using dcm2niix.^30^ The radioactivity concentrations in the PET images were decay corrected to the point of tracer injection using a ^18^F half-life of 109.77 min, and values of activity were converted into standard uptake values (SUV) according to equation 1 below:

$SUV=PET image\times\frac{Body weight}{Injected dose}\times e^{\frac{ln2}{t_{\frac{1}{2}}}(Time interval)}$ *Equation 1*

where *SUV* are in units of g mL^-1^, the *PET image* is represented by the values of activity in the PET image field of view and is in units of Bq mL^-1^, *Body weight* is in units of g, *Injection dose* is in units of Bq, *t_1/2_* is the radiotracer half-life and is in units of min, *Time interval* is the time passed between the injection of the radiotracer and the PET scan acquisition and is in units of min. ^18^F-FET PET images were then registered using 3D Euler rigid transformations (six degrees of freedom) to the CT image (optimizer: gradient descent, similarity metric: mattes mutual information) followed by linear resampling (interpolator: trilinear) to the CT image resolution. A binary mask of the brain tissue was manually drawn from the overlay of the ^18^F-FET PET and the CT image. The brain mask was applied to the ^18^F-FET PET image to obtain the ^18^F-FET PET brain-extracted image.

**Supplementary figures and tables**


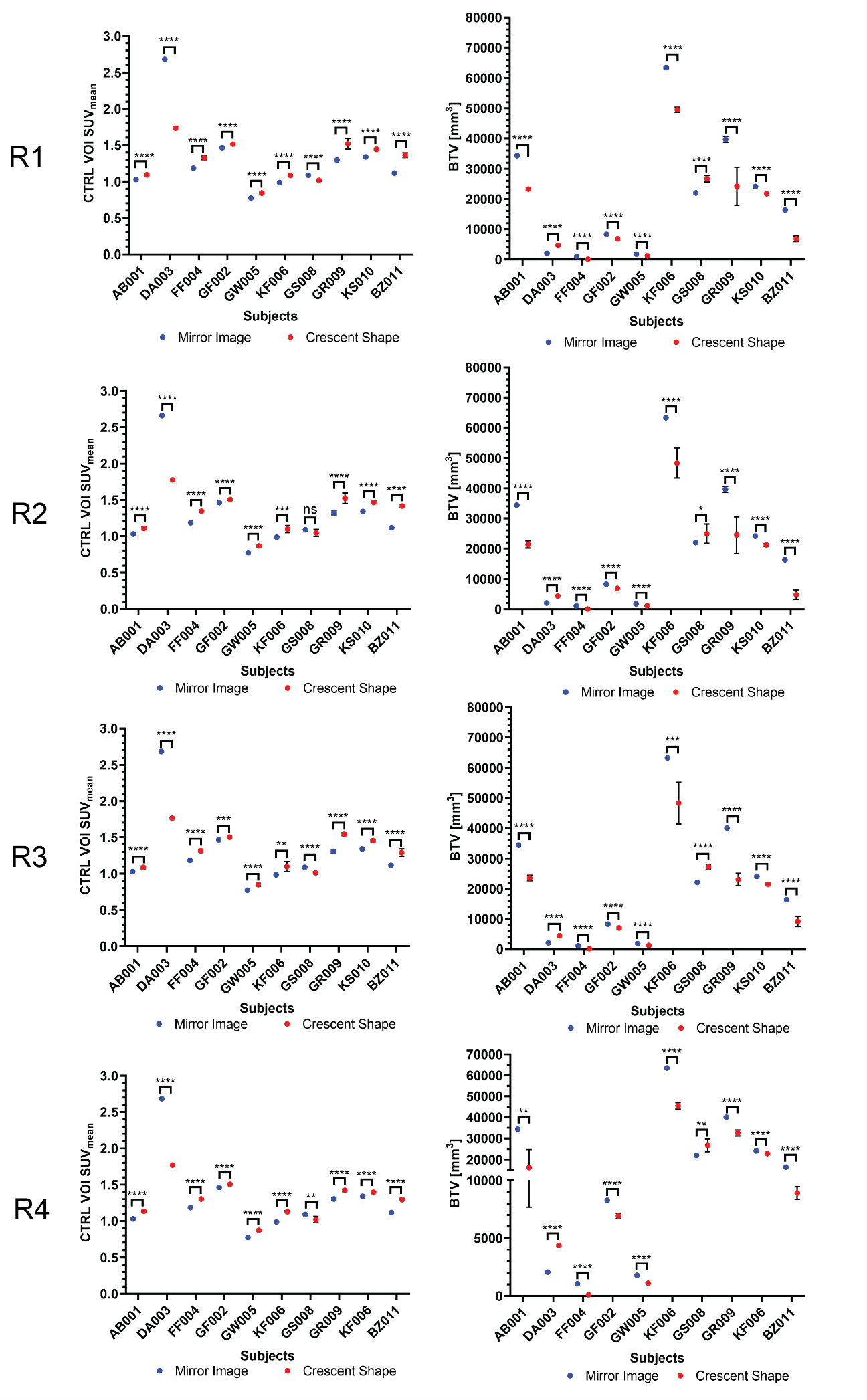


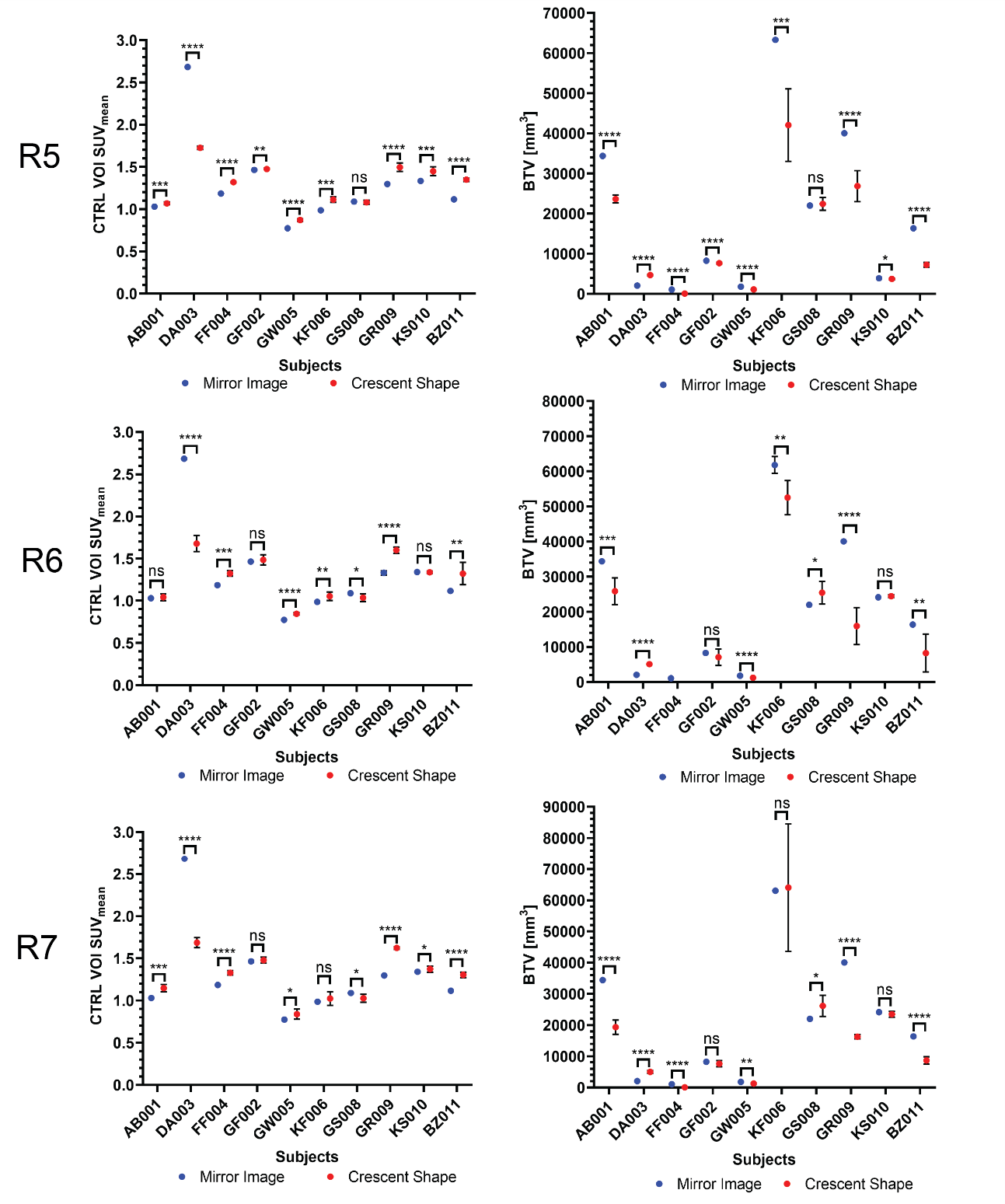


**Fig. S1** *Individual values of CTRL SUV_mean_ and BTV volume per reader*. The plots show the comparison of mean values of CTRL SUV_mean_ (left column) and BTV (right column) between the two methods obtained by each reader (R#) for all the datasets. The dot represents the mean value of the six repeats and the error bars represent the standard deviation. Multiple unpaired t test, Holm-Šídák method, α=0.05. * p < 0.05, ** p < 0.01, *** p < 0.001, **** p < 0.0001, ns = no significant difference. BTV = biological tumour volume; CTRL = contralateral background reference region; gCS = guided crescent-shape; MI = mirror-image; SUV = standard uptake value; VOI = volume of interest


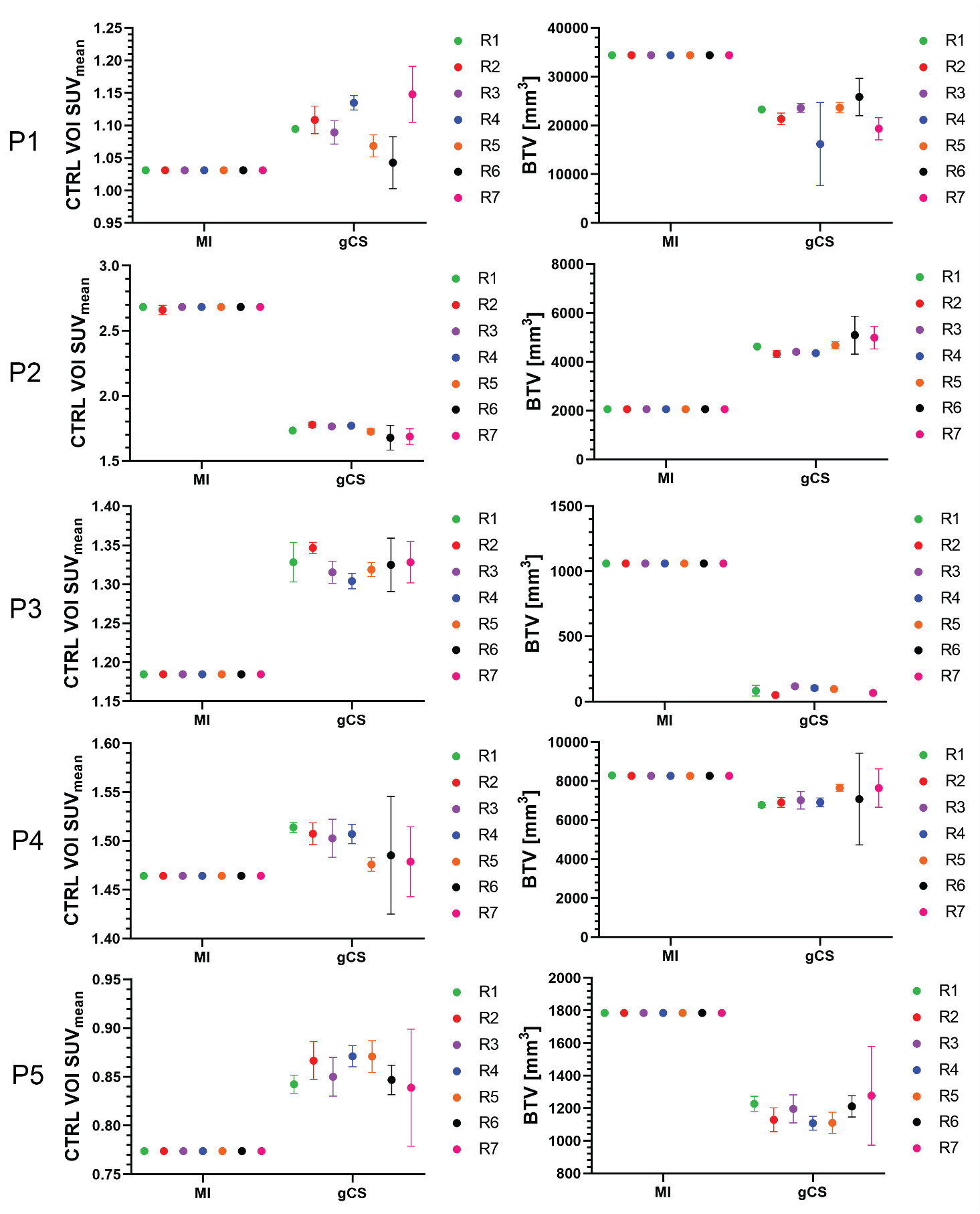


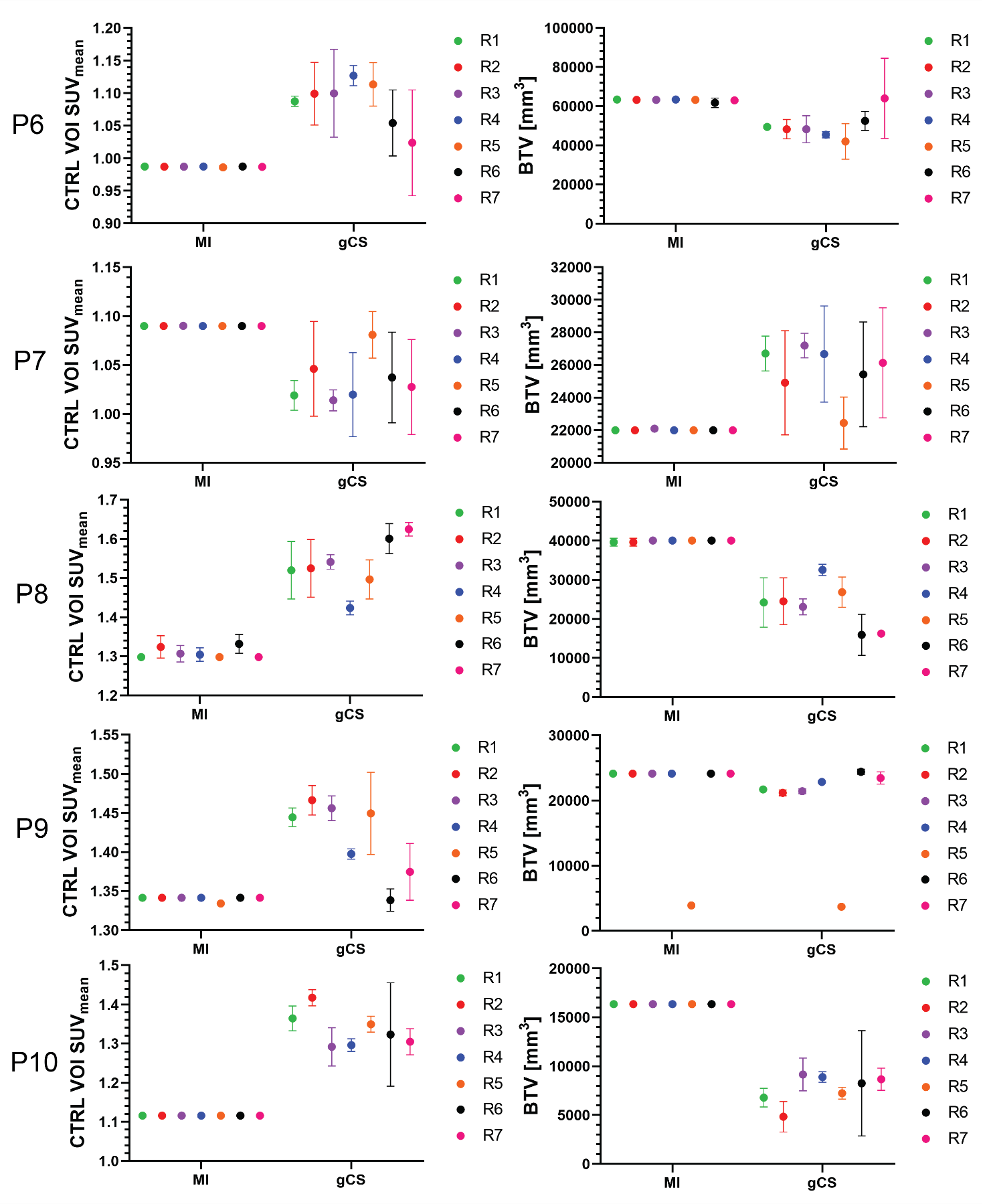


**Fig. S2** *Individual values of CTRL SUV_mean_ and BTV volume per patient dataset.* The plots show the comparison of mean values of CTRL SUV_mean_ (left column) and BTV (right column) between the two methods obtained for each patient dataset (P#) by all the readers (R#). The dot represents the mean value of the six repeats and the error bars represent the standard deviation. BTV = biological tumour volume; CTRL = contralateral background reference region; gCS = guided crescent-shape; MI = mirror-image; SUV = standard uptake value; VOI = volume of interest

**
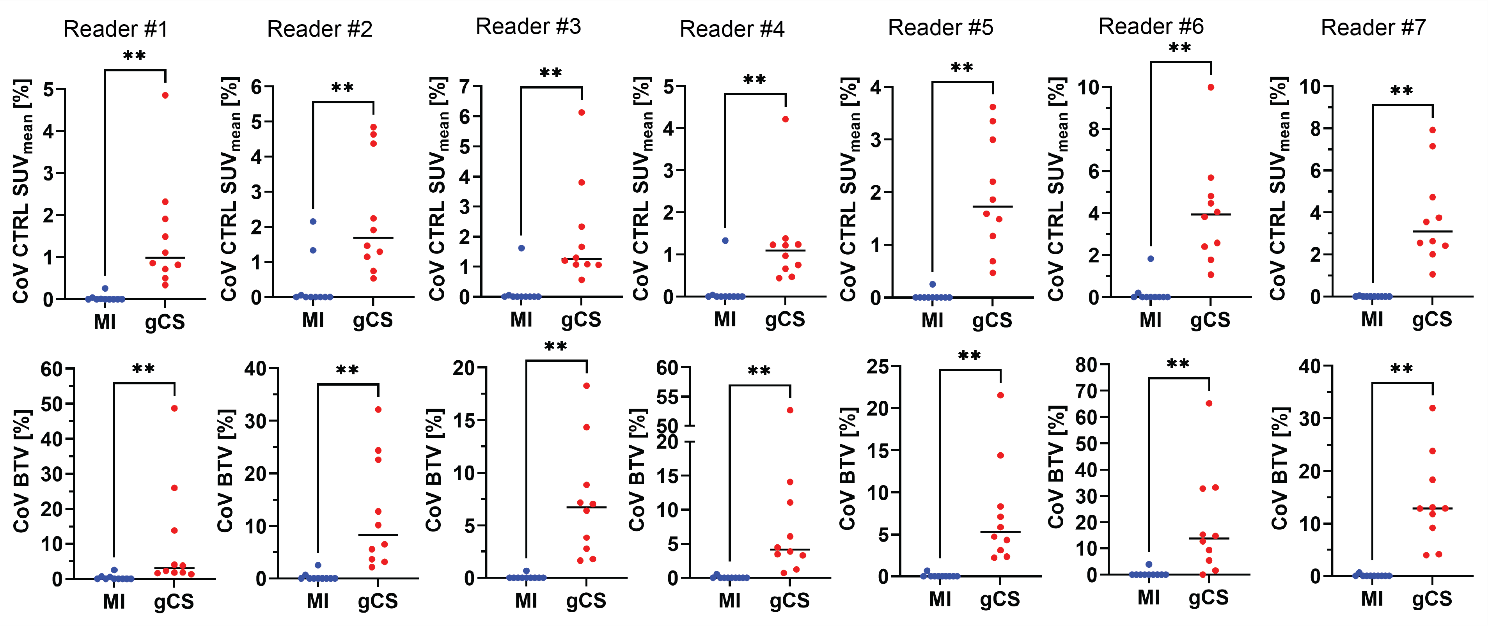
Fig. S3** *Summary plots of intra-reader coefficient of variation (CoV) for individual readers*. The plots show the comparison of the intra-reader CoV of the CTRL SUV_mean_ (top row) and BTV (bottom row) between the MI (blue) and the gCS (red) methods for the individual readers. Each point represents a single scan aggregated over reader repeat localizations. * p < 0.05, ** p < 0.01, *** p < 0.001, **** p < 0.0001, ns = no significant difference. BTV = biological tumour volume; CoV = coefficient of variation; CTRL = contralateral background reference region; gCS = guided crescent-shape; MI = mirror-image; SUV = standard uptake value; VOI = volume of interest


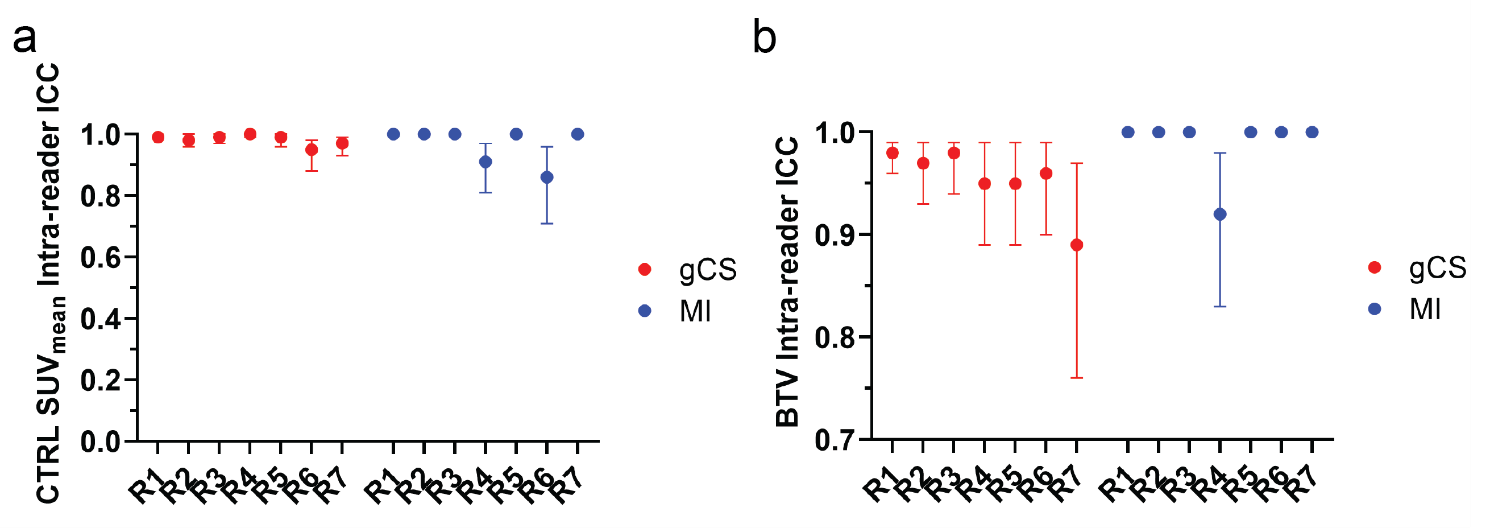


**Fig. S4** *Comparison of intra-reader intraclass correlation coefficients* of **a** CTRL SUV_mean_ and **b** BTV between the gCS and the MI methods. BTV = biological tumour volume; CTRL = contralateral background reference region; gCS = guided crescent-shape; ICC = intraclass correlation coefficient; MI = mirror-image; SUV = standard uptake value

**Table S1** Intra-reader intraclass correlation coefficients of CTRL SUV_mean_ and BTV volume^[[1]](#footnote-1)^

| **Metric** | **Method** | **Reader #** | **ICC** | **F** | **pval** | **CI 95%** |
| --- | --- | --- | --- | --- | --- | --- |
| **CTRL SUV_mean_** | gCS | 1 | 0.99 | 555 | <0.00001 | [0.98 1. ] |
|  |  | 2 | 0.98 | 383 | <0.00001 | [0.96 1. ] |
|  |  | 3 | 0.99 | 728 | <0.00001 | [0.97 1. ] |
|  |  | 4 | 1.00 | 1243 | <0.00001 | [0.99 1. ] |
|  |  | 5 | 0.99 | 926 | <0.00001 | [0.96 1. ] |
|  |  | 6 | 0.95 | 122 | <0.00001 | [0.88 0.98] |
|  |  | 7 | 0.97 | 261 | <0.00001 | [0.93 0.99] |
|  | MI | 1 | 1.00 | 1428721 | <0.00001 | [1. 1.] |
|  |  | 2 | 1.00 | 7907 | <0.00001 | [1. 1.] |
|  |  | 3 | 1.00 | 36980 | <0.00001 | [1. 1.] |
|  |  | 4 | 0.91 | 65 | <0.00001 | [0.81 0.97] |
|  |  | 5 | 1.00 | 2661632 | <0.00001 | [1. 1.] |
|  |  | 6 | 0.86 | 37 | <0.00001 | [0.71 0.96] |
|  |  | 7 | 1.00 | 69631631 | <0.00001 | [1. 1.] |
| **BTV** | gCS | 1 | 0.98 | 324 | <0.00001 | [0.96 0.99] |
|  |  | 2 | 0.97 | 190 | <0.00001 | [0.93 0.99] |
|  |  | 3 | 0.98 | 272 | <0.00001 | [0.94 0.99] |
|  |  | 4 | 0.95 | 115 | <0.00001 | [0.89 0.99] |
|  |  | 5 | 0.95 | 131 | <0.00001 | [0.89 0.99] |
|  |  | 6 | 0.96 | 124 | <0.00001 | [0.90 0.99] |
|  |  | 7 | 0.89 | 49 | <0.00001 | [0.76 0.97] |
|  | MI | 1 | 1.00 | 21883 | <0.00001 | [1. 1.] |
|  |  | 2 | 1.00 | 20373 | <0.00001 | [1. 1.] |
|  |  | 3 | 1.00 | 142270 | <0.00001 | [1. 1.] |
|  |  | 4 | 0.92 | 73 | <0.00001 | [0.83 0.98] |
|  |  | 5 | 1.00 | 152219 | <0.00001 | [1. 1.] |
|  |  | 6 | 1.00 | 3671 | <0.00001 | [1. 1.] |
|  |  | 7 | 1.00 | 141394 | <0.00001 | [1. 1.] |

**Table S2** Inter-reader intraclass correlation coefficients of CTRL SUV_mean_ and BTV^1^

| **Metric** | **Method** | **ICC** | **F** | **pval** | **CI 95%** |
| --- | --- | --- | --- | --- | --- |
| **CTRL SUV_mean_** | gCS | 0.98 | 367 | <0.00001 | [0.96 0.99] |
|  | MI | 1.00 | 68454 | <0.00001 | [1. 1.] |
| **BTV** | gCS | 0.94 | 106 | <0.00001 | [0.87 0.98] |
|  | MI | 0.99 | 476 | <0.00001 | [0.97 1. ] |

1. BTV = biological tumour volume; SUV = standard uptake value; gCS = guided crescent-shape; MI = mirror-image; ICC = intraclass correlation coefficient; F = F test with true value 0; CI 95% = 95% confidence interval. [↑](#footnote-ref-1)
